# Feasibility of Reduced Iodine Loads for Vascular Assessment Prior to Transcatheter Aortic Valve Implantation (TAVI) Using Spectral Detector CT

**DOI:** 10.3390/diagnostics14090879

**Published:** 2024-04-24

**Authors:** Christopher Schuppert, Janek Salatzki, Florian André, Johannes Riffel, David L. Mangold, Claudius Melzig, Muhammad Taha Hagar, Hans-Ulrich Kauczor, Tim F. Weber, Fabian Rengier, Thuy D. Do

**Affiliations:** 1 Department of Diagnostic and Interventional Radiology, Medical Center—University of Freiburg, Faculty of Medicine, University of Freiburg, 79106 Freiburg im Breisgau, Germany; 2 Clinic of Diagnostic and Interventional Radiology, Heidelberg University Hospital, 69120 Heidelberg, Germany; 3 Clinic of Cardiology, Angiology and Pneumology, Heidelberg University Hospital, 69120 Heidelberg, Germany; 4 Department of Cardiology and Angiology, Robert Bosch Hospital, 70376 Stuttgart, Germany

**Keywords:** transcatheter aortic valve implantation, transcatheter aortic valve replacement, cardiovascular imaging, dual-layer detector CT, spectral detector CT, contrast media

## Abstract

Reduced iodine loads for computed tomography (CT)-based vascular assessment prior to transcatheter aortic valve implantation (TAVI) may be feasible in conjunction with a spectral detector CT scanner. This prospective single-center study considered 100 consecutive patients clinically referred for pre-TAVI CT. They were examined on a dual-layer detector CT scanner to obtain an ECG-gated cardiac scan and a non-ECG-gated aortoiliofemoral scan. Either a standard contrast media (SCM) protocol using 80 mL Iohexol 350 mgI/mL (iodine load: 28 gI) or a body-mass-index adjusted reduced contrast media (RCM) protocol using 40–70 mL Iohexol 350 mgI/mL (iodine load: 14–24.5 gI) were employed. Conventional images and virtual monoenergetic images at 40–80 keV were reconstructed. A threshold of 250 HU was set for sufficient attenuation along the arterial access pathway. A qualitative assessment used a five-point Likert scale. Sufficient attenuation in the thoracic aorta was observed for all patients in both groups using conventional images. In the abdominal, iliac, and femoral segments, sufficient attenuation was observed for the majority of patients when using virtual monoenergetic images (SCM: 96–100% of patients, RCM: 88–94%) without statistical difference between both groups. Segments with attenuation measurements below the threshold remained qualitatively assessable as well. Likert scores were ‘excellent’ for virtual monoenergetic images 50 keV and 55 keV in both groups (RCM: 1.2–1.4, SCM: 1.2–1.3). With diagnostic image quality maintained, it can be concluded that reduced iodine loads of 14–24.5 gI are feasible for pre-TAVI vascular assessment on a spectral detector CT scanner.

## 1. Introduction

Patients planned for transcatheter aortic valve implantation/replacement (TAVI/TAVR) routinely undergo computed tomography (CT) angiography [1,2]. An integral part of the pre-interventional workup, this examination enables the assessment of the anatomy of the aortic valvular complex and the arterial access pathway including patency and tortuosity [3,4]. However, it requires intravenous application of iodinated contrast media (CM) in a multimorbid population of advanced age with a high prevalence of impaired renal function and, consequently, elevated risk of contrast-associated nephropathy [5,6]. Additional CM will have to be administered during the TAVI procedure itself. Acute kidney injury is a recognized complication following TAVI, and new post-interventional renal replacement therapy is associated with an increased one-year mortality, independent of the access site [7]. Therefore, efforts in lowering the iodine load required for pre-TAVI imaging are highly relevant, yet difficult to accomplish without compromising the image quality.

Past efforts used low tube potential CT acquisition protocols (70–80 kV_p_) to enhance iodine attenuation in CM-reduced scans; however, these may come at the cost of exceedingly high image noise in obese patients [8]. An alternative approach leverages spectral detector CT and virtual monoenergetic images (VMI, also virtual monochromatic images) [9,10]. Spectral detector CT uses two separate detectors mounted on top of each other (dual-layer detector, DLCT). They absorb X-ray photons at different energy levels, thus enabling the retrospective extraction of spectral data and VMI to maintain image quality [11]. The potential of DLCT and VMI for allowing reduced CM volumes and iodine loads in pre-TAVI imaging has yet to be assessed.

Recognizing this opportunity, the purpose of this study was to determine the feasibility of reduced iodine loads for vascular assessment prior to transcatheter aortic valve implantation in conjunction with a DLCT scanner.

## 2. Material and Methods

### 2.1. Study Sample

**Figure 1:**
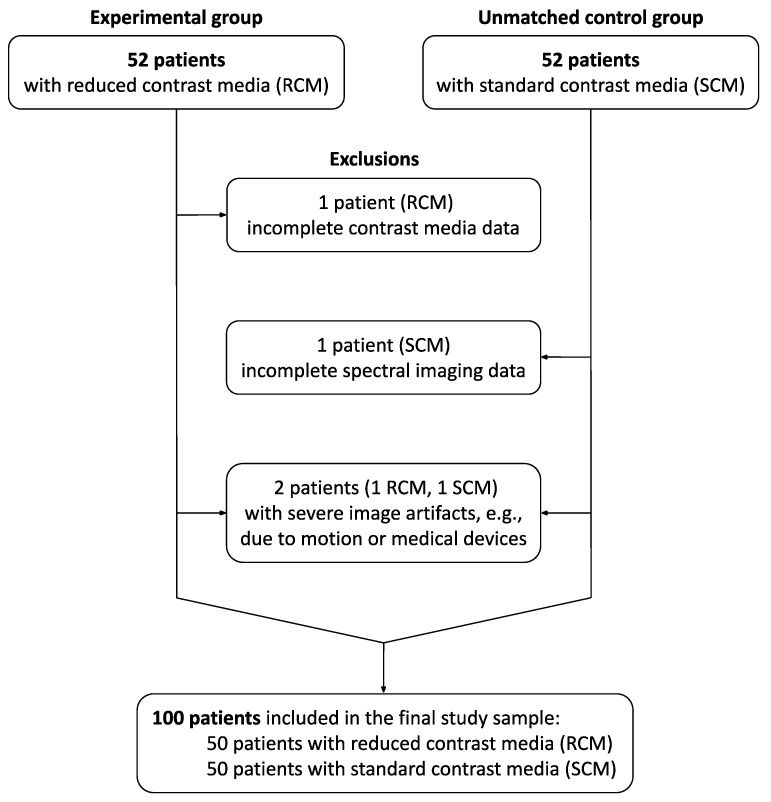
Flow chart of the inclusion and exclusion process.

Patients referred to pre-TAVI CT imaging were asked to participate in a prospective study evaluating reduced dosages of intravenous CM with adjustment to the body mass index (BMI) on a DLCT scanner. Inclusion criteria were indication for aortic valve replacement according to the present guidelines from the European Society of Cardiology (ESC) and the European Association for Cardio-Thoracic Surgery (EACTS) [12], attainment of at least 18 years, and the ability to give consent. Exclusion criteria were previous anaphylactic-type reactions to iodinated CM or decline to participate. From this prospective cohort, we included 52 consecutive patients examined between July 2020 and September 2020 (RCM group). As an unmatched control group, we included 52 consecutive patients examined with the standard departmental CM dosage between November 2020 and January 2021 (SCM group). Further exclusion criteria regarding CT image quality were severe image artifacts, such as those from bulk patient motion or medical devices, incomplete spectral imaging data for post-processing, CM extravasation at the injection site, or incomplete CM injection data. The final study sample comprised 50 patients in the RCM group and 50 patients in the SCM group (Figure 1, Table 1). In this sample, 87 patients underwent an aortic valve procedure: TAVI through a transfemoral (*n* = 77), transaortic (*n* = 1), or transapical (*n* = 6) approach, balloon valvuloplasty (*n* = 1), or surgical aortic valve replacement (*n* = 2). For these patients, post-procedural (last test before discharge) serum creatinine levels were collected from the records to complement the serum creatinine levels used for pre-imaging renal function assessment. Estimated glomerular filtration rates (eGFR) were calculated according to the 2021 CKD-EPI creatinine equation [13].

### 2.2. DLCT Image Acquisition Protocol

CT imaging was performed using a dual-layer detector spectral system (IQon Spectral CT, Philips Healthcare, Amsterdam, The Netherlands). The split protocol comprised a low-pitch cardiac scan with retrospective ECG-gating, acquired in the caudocranial direction, and a subsequent non-ECG-gated high-pitch aortoiliofemoral scan, acquired in the craniocaudal direction during the late arterial phase. The acquisition parameters for the cardiac and aorta scans were as follows: beam collimation 64 × 0.625 mm, peak tube potential 120 kV_p_, average tube current-time product 600 and 130 mAs with a predicted volumetric computed tomography dose index (CTDI_vol_) of 54.2 and 11.8 mGy (32 cm body-phantom), dose right index 18 (automated, attenuation-based dose modulation), gantry rotation time 0.27 s and 0.4 s, pitch 0.2 and 1.1234 (Table 2). The field of view (FOV) was variable depending on the body volume. The measured CTDI_vol_ and dose length product (DLP) were noted.

The cardiac scan was triggered using bolus tracking with an ROI in the ascending aorta 6 s after reaching a threshold of 110 Hounsfield units (HU), and the body scan started with a delay of 26 s after the threshold value. Both scans were acquired in inspiratory breath hold. This split protocol was chosen based on expert consensus for a detector coverage of 4 cm and a previous retrospective study [3,14,15].

### 2.3. Contrast Media Injection Protocols

Nonionic low-osmolar iodinated CM with a concentration of 350 mgI/mL Iohexol (Accupaque 350, GE Healthcare, Chicago, IL, USA) was administered via antecubital venous access using a dual-syringe power injector (MEDRAD Stellant, Bayer Vital GmbH, Leverkusen, Germany). The injected CM volume in the SCM group was 80 mL, whereas in the RCM group, it was adjusted to BMI based on the existing literature [16,17] and the findings of in-house testing using an aorta phantom (Philips Healthcare, Amsterdam, The Netherlands) with varied volumes of 350 mgI/mL CM starting at 80 mL and subsequent reductions in steps of 10 mL. The CM volumes decided upon for the RCM group were 40 mL for BMI < 23 kg/m^2^, 50 mL for BMI 23–30 kg/m^2^, 70 mL for BMI > 30 kg/m^2^. In either group, an initial injection of 40 mL at 4 mL/s (SCM group: 100% CM, RCM group: 50% CM diluted with saline for BMI < 23 kg/m^2^, 75% for BMI 23–30 kg/m^2^ and BMI > 30) was immediately followed by 40 mL at 3 mL/s (SCM group: 100% CM, RCM group: 50% CM diluted with saline for BMI < 23 kg/m^2^, 50% for BMI 23–30 kg/m^2^, 100% for BMI > 30) and a saline flush of 50 mL at 3 mL/s to form a single bolus injection. The total iodine loads were 28 gI for the SCM group and 14 gI, 17.5 gI, or 24.5 gI for the RCM group. Current guidelines were followed for preventive hydration and management of metformin therapy [18].

### 2.4. Image Reconstruction

**Figure 2:**
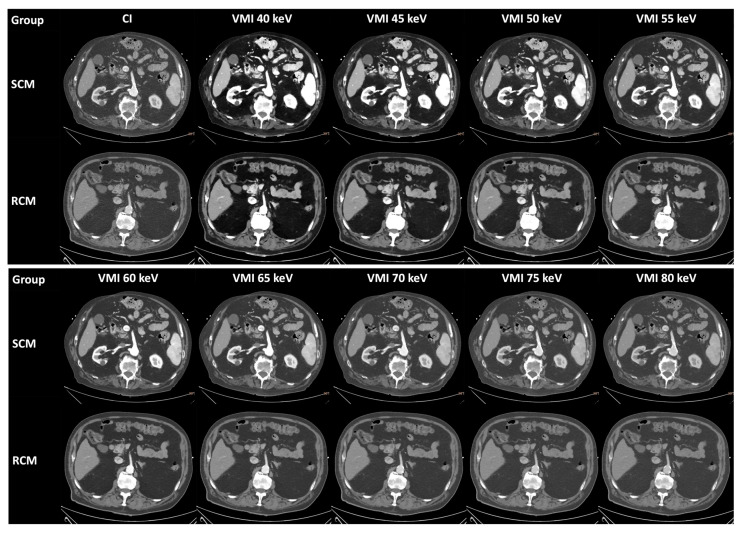
Conventional images and virtual monoenergetic images from both groups. Example images from both groups were taken from the aortoiliofemoral scans and show the abdominal aorta at the height of the superior mesenteric artery at different energy levels: 40 keV to 80 keV in 5 keV intervals. The two patients from the SCM group and the RCM group had a BMI in the 23–30 range. Window level: 50 HU. Window width: 600 HU. VMI: virtual monoenergetic images. SCM: standard contrast media volume. RCM: reduced contrast media volume. HU: Hounsfield unit.

DLCT data were reconstructed using the manufacturer’s dedicated post-processing software IntelliSpace Portal (version 12, Philips Healthcare, Best, The Netherlands). Aside from conventional images (CI), VMI were reconstructed from 40 keV to 80 keV in increments of 5 keV (Figure 2). All reconstructions were performed in axial orientation with a slice thickness of 3 mm, increment of 1.5 mm, soft-tissue kernel, and used an iterative reconstruction algorithm (IMR Level 1). The cardiac scans were reconstructed in best systolic phase and in diastole at 75% of the R-R interval, of which the diastolic series were used for the following analyses of the arterial access pathway.

### 2.5. Image Analysis

**Figure 3:**
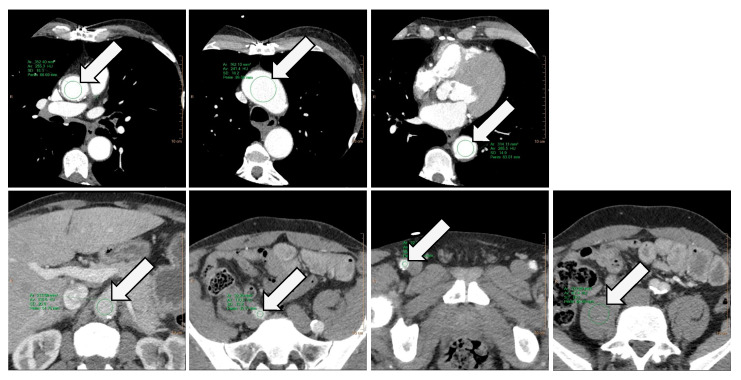
Regions of interest for quantitative data collection. ROIs were placed for the measurement of mean attenuation, signal-to-noise ratio, and contrast-to-noise ratio. The cardiac scan was used for three ROIs in the ascending aorta, the aortic arch, and the descending aorta (arrows; upper row), whereas the aortoiliofemoral scan was used for four ROIs in the abdominal aorta at the height of the superior mesenteric artery, the right common iliac artery, the right common femoral artery, and the right psoas muscle (arrows; lower row). ROI: region of interest.

Quantitative image assessment was performed in IntelliSpace Portal by placing circular intravascular regions of interest (ROI) at six levels of the arterial access pathway: aortic root, aortic arch, descending thoracic aorta at the height of the cardiac valves, abdominal aorta at the height of the superior mesenteric artery, right common iliac artery, and right common femoral artery (Figure 3). An extravascular ROI was placed in the right psoas muscle for CNR evaluation. The cardiac scan was used for the three ROIs covering the thoracic aorta, and the thoracoabdominal scan was used for the remaining four ROIs. All ROIs were placed as large as possible while carefully avoiding arteriosclerotic calcification plaques. Attenuation values and noise were noted as mean and standard deviation (ROI_mean_ and ROI_sd_). Signal-to-noise ratios (SNR) were calculated as VesselROI_mean_: VesselROI_sd_, and contrast-to-noise ratios (CNR) as (VesselROI_mean_ − PsoasROI_mean_): VesselROI_sd_. Sufficient mean vessel attenuation was defined as ≥250 HU at all levels according to recent recommendations [3,19].

Qualitative image assessment was performed in IntelliSpace Portal on clinically used workstations by two board-certified readers with nine years (Reader I) and six years (Reader II) of work experience in radiology. They rated the diagnostic quality of the vessel depiction along the arterial access pathway on CI and VMI at 50 and 55 keV using a five-point Likert scale (1: excellent, 2: good, 3: fair, 4: poor [but barely sufficient], 5: non-diagnostic). The cardiac scan was considered for the ascending to descending aorta, and the aortoiliofemoral scan was considered for the abdominal aorta to the femoral arteries. Window adjustments were allowed. The VMI energy levels were chosen based on the results of the qualitative analysis with the lowest noise and highest possible SNR and CNR.

### 2.6. Statistics

Statistical analysis was conducted using SAS (version 9.4, SAS Institute Inc., Cary, NC, USA). Normal distribution of the data was tested using the Shapiro–Wilk test. Descriptive statistics of patient characteristics, quantitative image quality parameters, radiation dose measures, and pre-imaging/post-procedural delta of creatinine levels were determined using mean and standard deviation for continuous variables with normal distribution, median and (interquartile) range for continuous variables with non-normal distribution, and percentages for categorical variables. Inter-group comparisons of these variables used the two-sided unpaired Student’s t-test for normally distributed data, the Mann–Whitney U test for non-normally distributed data, and the Chi-squared test of independence for categorical variables. Sufficient attenuation was additionally assessed on a per-patient level using counts and percentages. Intra-individual comparisons of attenuation values were performed for CI against VMI at 50 and 55 keV and used the Wilcoxon signed-rank test. The qualitative image quality ratings were compared using mean values as well as the worst score counts principle. Their differences were tested using the Mann–Whitney U test for independent and the Wilcoxon signed-rank test for dependent samples. Inter-rater variability was assessed using percentage agreement. The level of statistical significance was set at *p* < 0.05.

### 2.7. Ethical Approval and Patient Consent

This study conformed to the ethical guidelines of the 1964 Declaration of Helsinki and its later amendments. Written informed consent was obtained from patients in the prospective cohort and waived for patients in the retrospective control group. The study was approved by the Ethics Committee of the Medical Faculty of Heidelberg University, Germany (Ref. S-678/2018).

## 3. Results

### 3.1. Patient Characteristics

The patients in both groups did not differ significantly regarding gender (*p* = 0.03), age (*p* = 0.06), or BMI (*p* = 0.36). The median interval between CT imaging and the aortic valve procedure was 9 days (IQR: 1–33).

### 3.2. Quantitative Image Quality Assessment in Conventional Images

**Figure 4:**
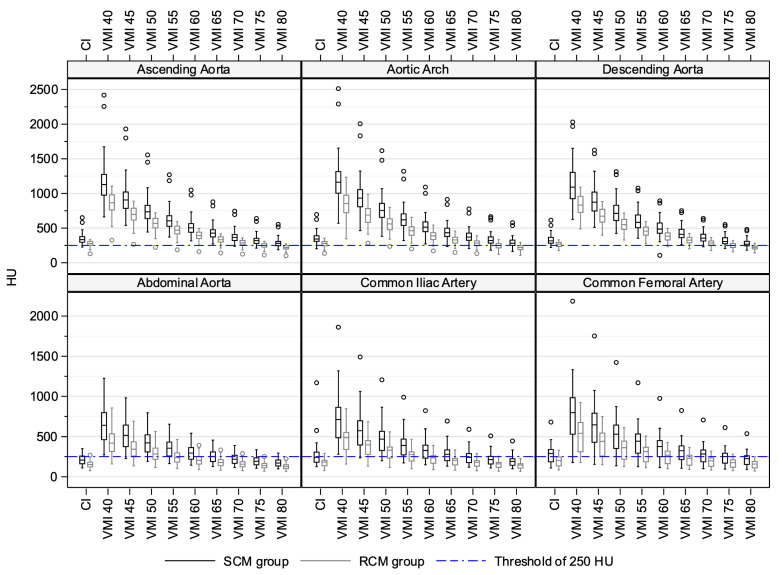
Attenuation values from CI and VMI at energy levels of 40 keV to 80 keV in steps of 5 keV. Data are presented as Box–Whisker Plots. Vessel segments were considered individually. CI: conventional images. VMI: virtual monoenergetic images. SCM: standard contrast media volume. RCM: reduced contrast media volume.

Sufficient attenuation in the thoracic segments was found for all patients. In contrast, sufficient attenuation in abdominal, iliac, and femoral segments was observed for 15 (30%), 22 (44%), and 33 (66%) patients in the SCM group, compared to 1 (2%), 7 (14%), and 11 (22%) patients in the RCM group, respectively (*p* = 0.01). The median attenuation for all vessel segments was consistently lower in the RCM group than the SCM group within a range of 47–87 HU or 14.9–30.0% (all *p* < 0.001) (Table 3, Figure 4). At the same time, a distinct attenuation pattern along the direction of flow was retained in both groups: a steplike decrease along the three thoracic segments was followed by a stepwise increase along the three abdominofemoral segments.

**Figure 5:**
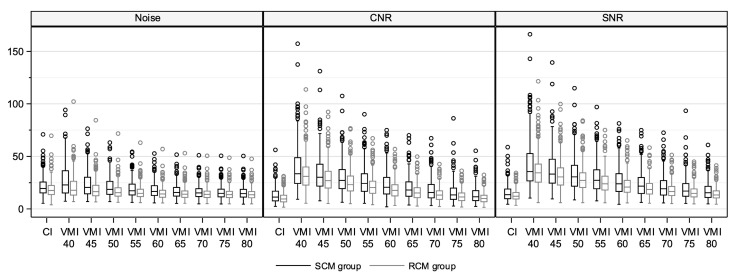
Noise, CNR, and SNR from CI and VMI at energy levels of 40 keV to 80 keV in steps of 5 keV. Data are presented as Box–Whisker Plots. All six vessel segments were considered together. CI: conventional images. VMI: virtual monoenergetic images. SCM: standard contrast media volume. RCM: reduced contrast media volume.

The median image noise across all vessel segments remained constant or decreased slightly in the RCM group, as did the median CNR and SNR (Table 3, Figure 5). Statistically significant differences for CNR and SNR were only observed for the three abdominofemoral segments with a delta range of 3–8 (*p* = 0.001, *p* = 0.01, *p* = 0.003) for CNR and 1–5 (*p* = 0.002, *p* = 0.03, *p* = 0.03) for SNR, respectively.

### 3.3. Quantitative Image Quality Assessment in Virtual Monoenergetic Images

All reconstructed energy levels taken into consideration, sufficient attenuation in the thoracic vessel segments was found for all patients, and for the majority of patients regarding the abdominal, iliac, and femoral vessel segments (RCM: 88%, 88%, and 94%, SCM: 100%, 100%, and 96%). This finding was without significant difference between both groups (*p* = 0.3). The main difference of VMI findings to CI findings, therefore, concerned the abdominal vessel segments, which in CI from both groups mostly failed to fulfill the quantitative attenuation requirement. This was likewise apparent in the median attenuation values of vessel segments across patients, as these also stayed above the accepted threshold of 250 HU at selected VMI energy levels, contrary to the corresponding median attenuation values in CI (Figure 4). For the RCM group, depending on the specific abdominal vessel segment, this was achieved in energy levels of 40 keV to 50–60 keV. For both groups and all vessel segments, the median attenuation decreased with increasing keV, thereby following the mass attenuation coefficient of iodine, and was higher than in CI at multiple energy levels. In an observation similar to CI, attenuation in each vessel segment was consistently lower in the RCM group than in the SCM group (all *p* < 0.001) and followed a decrease–increase pattern along the direction of flow (Table 3, Figure 4).

The lowest noise levels in combination with the highest possible SNR and CNR were found in VMI 50 keV and 55 keV (Figure 5). For these two energy levels, the median attenuation differences to CI are given in Table 4. The attenuation differences between CI and VMI were smallest at 70 keV.

### 3.4. Qualitative Image Quality Assessment

**Figure 6:**
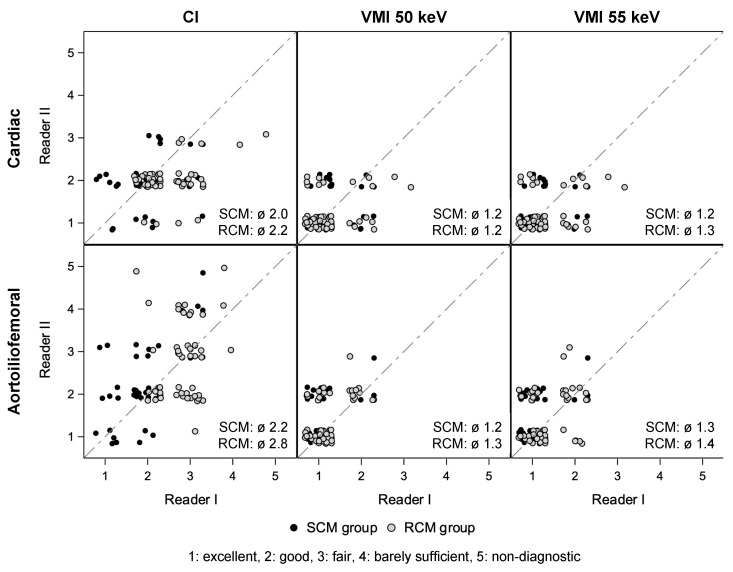
Qualitative ratings from two readers using a five-point Likert scale. The qualitative assessment comprised CI and VMI at 50 keV and 55 keV from the cardiac and aortoiliofemoral scans and was performed for both groups. Data are presented as scatter plots. ‘Jittering’ avoids overlying data points. CI: conventional images. VMI: virtual monoenergetic images. SCM: standard contrast media volume). RCM: reduced contrast media volume.

The inter-rater agreement was acceptable at 65% and improved to 86% when the ratings ‘good’ and ‘excellent’ were considered as one (Figure 6). CI from the cardiac and aortoiliofemoral scans received averaged ratings of 2.0 and 2.2 in the SCM group and significantly poorer ratings of 2.2 and 2.8, respectively, in the RCM group (*p* = 0.03 and *p* = 0.001). VMI at 50 keV and 55 keV averaged 1.2–1.3 in the SCM group and 1.2–1.4 in the RCM group without statistically significant differences between the groups (50 keV: *p* = 0.68 and *p* = 0.08 for the cardiac and aortoiliofemoral scans, 55 keV: *p* = 0.37 and *p* = 0.02, respectively). The differences in quality ratings between 50 keV and 55 keV were mostly not significant (RCM: *p* = 0.29 and *p* = 0.01 for the cardiac and aortoiliofemoral scans, SCM: *p* = 0.99 and *p* = 0.22, respectively).

In the worst score counts analysis, the quality ratings ‘poor’ and ‘non-diagnostic’ were limited to CI and distributed differently between the groups: In the SCM group, only the aortoiliofemoral scan was affected (14% of 50 patients), whereas the RCM group showed higher frequencies of these ratings in both the cardiac scan (4%) and the aortoilio-femoral scan (26%). However, each affected series improved to ‘fair’, ‘good’, or ‘excellent’ ratings in VMI at 50 and 55 keV.

### 3.5. Radiation Dose

The median CTDI_vol_ and DLP for the combined scans were 9.6 mGy (IQR: 8.5–11.2) and 660 mGy*cm (IQR: 593–740) in the SCM group compared to 9.3 mGy (IQR: 8.2–10.6) and 642 mGy*cm (IQR: 548–754) in the RCM group. These differences were not statistically significant at *p* = 0.58 and *p* = 0.45, respectively.

### 3.6. Renal Function

The median interval between the pre-imaging serum creatinine assessment and CT was 1 day (IQR: 0–6), compared to 20 days (IQR: 7–38) between CT and the post-procedural serum creatinine assessment. The median pre-imaging and post-procedural serum creatinine levels were 1.01 mg/dL (IQR: 0.82–1.41) and 1.01 mg/dL (IQR: 0.89–1.24) in the SCM group (*n* = 43) compared to 0.95 mg/dL (IQR: 0.78–1.29) and 0.90 mg/dL (IQR: 0.75–1.31) in the RCM group (*n* = 44). The corresponding eGFR were 66.0 mL/min/1.73 m^2^ (IQR: 43.9–81.0) and 64.4 mL/min/1.73 m^2^ (IQR: 52.5–76.9) compared to 62.5 mL/min/1.73 m^2^ (IQR: 44.4–80.8) and 67.5 mL/min/1.73 m^2^ (IQR: 45.8–81.4). The intra-individual differences between serum creatinine levels were not statistically significant at *p* = 0.59.

## 4. Discussion

We evaluated prospective study data to determine the feasibility of reduced iodine loads for vascular assessment prior to transcatheter aortic valve implantation (TAVI) in conjunction with a dual-layer detector CT (DLCT) scanner. In two groups of 50 patients each, either a standard dosage of iodinated contrast media (CM) (80 mL of 350 mg iodine/mL, 28 gI) or a BMI-adjusted reduced dosage (40–70 mL of 350 mg iodine/mL, 14–24.5 gI) was applied. Our quantitative and qualitative analysis revealed that sufficient image quality for vascular assessment could be achieved in the reduced CM group using virtual monoenergetic images.

Preceding investigations on reducing iodine loads in pre-TAVI CT imaging on multidetector-row scanners referenced using between 110 and 140 mL of 320 mgI/mL or 350 mgI/mL CM (35.2–49.0 gI) [20,21]. Following the introduction of second-generation dual-source scanners capable of prospectively ECG-triggered high-pitch spiral acquisitions, Wuest et al. successfully validated using 40 mL of 350 mgI/mL CM on 42 patients (iodine load without test bolus: 14 gI) [22]. In a subsequent study, Azzalini et al. reduced this further to 15 mL of 370 mgI/mL CM (iodine load without test bolus: 5.6 gI) and still found an image quality suitable for pre-TAVI assessment [23]. Both studies did not include a retrospectively ECG-gated scan, whose ability for the dynamic assessment of the aortic valve and annulus has since been found advantageous for prosthesis planning [24]. Kok et al., therefore, tested a combined scan protocol with a retrospective ECG-gated cardiac CT at 80 kV_p_ followed by a non-gated high-pitch CT angiography at 70 kV_p_ (BMI < 28) or 80 kV_p_ (BMI > 28), additionally taking advantage of the increased iodine attenuation at low tube potentials. They found 40 mL or 53 mL (same stratification) of 300 mgI/mL CM (iodine loads without test bolus: 12 gI and 15.9 gI) to be sufficient for pre-interventional vascular assessment in a prospective study on 47 patients [8]. While low tube potentials restrict image quality by raising image noise and lowering soft-tissue contrast, amplified further in obese patients due to more absorbing tissue, increased levels of these side effects may be acceptable within the context of examinations focused on the pre-interventional evaluation of the arterial access pathway.

A more recent approach for the reduction of iodinated CM in pre-TAVI imaging utilizes VMI acquired from spectral detector CT scanners: Cavallo et al. prospectively studied 116 patients who underwent an ECG-gated chest scan followed by non-ECG-gated abdominopelvic scan that were performed using 50 mL of 350 mgI/mL CM (iodine load: 17.5 gI) on a DLCT scanner. They found that VMI at 40 keV provided sufficient quantitative and qualitative image quality for pre-TAVI assessment [10]. Similarly, Mangold et al. observed that using a single bolus of 80–100 mL of 400 mgI/mL CM (iodine load: 32–40 gI) with a split protocol comprising a retrospectively ECG-gated low-pitch cardiac scan plus a non-ECG-gated high-pitch aortoiliofemoral scan on a DLCT scanner combined with VMI at 40 keV led to superior image quality for pre-TAVI assessment compared to using the same CM amounts in a single retrospectively ECG-gated cardiac and aortoiliofemoral scan on a single-detector CT scanner that provided only CI at either 100 kV_p_ or 120 kV_p_ [15]. Their retrospective study, based on the quantitative assessment of attenuation, noise, CNR, and SNR in examinations from 150 patients, did not include a qualitative rating. Another recent study by Higashigaito et al. compared pre-TAVI examinations acquired on a photon-counting detector CT scanner with 52.5–70 mL of 370 mgI/mL CM (iodine load: 19.4–25.9 gI) to those from a third-generation CT scanner in conventional single-source mode with automated tube voltage selection and 70 mL of the same CM (iodine load: 25.9 gI). They demonstrated non-inferior image quality using the reduced CM dosage of 52.5 mL when using VMI at 50 keV [25].

Our study on reduced iodine loads for pre-TAVI vascular assessment follows up on these works and used 14–24.5 gI with BMI adjustment for examinations on a dual-layer detector CT scanner. The ability to reconstruct images from spectral data using this setup generally compensates for the main disadvantage of conventional low-keV acquisitions at reduced CM dosages, namely increased noise levels in the only available image series. In addition to the conventional image series, the reader is provided with VMI at different energy levels, each with a distinct distance from the k-edge of iodine at 33 keV. The image series with the best attenuation, noise, SNR, and CNR can then be retrospectively identified. In our quantitative assessment, favorable constellations of these combined parameters were found in VMI 50 keV and 55 keV. Using these reconstructions, attenuation was almost exclusively measured as >250 HU considering all vessel segments from both groups and CNR stayed >10. They were thus able to compensate for the poor quantitative characteristics of CI in the abdominal vessel segments. This important finding was confirmed in the qualitative assessment, where the ratings ‘poor’ and ‘non-diagnostic’ were limited to CI and primarily appeared in the RCM group, yet were all remedied in the corresponding VMI at 50 and 55 keV. It was further demonstrated that the SCM group benefitted from VMI as well, and there was no observable reader preference between the groups at these energy levels. The observed susceptibility of the abdominal vessel segments to low attenuation can in part be attributed to employing a single-bolus CM injection protocol in conjunction with a split image acquisition that places the aortoiliofemoral scan in the late arterial phase. While this combination had already been validated by Mangold et al. for standard CM dosages [15], a split-bolus injection may have been advantageous, especially for the BMI-adjusted reduced dosages.

The renal function did not decrease after pre-TAVI imaging in either group. Additionally, the results illustrate an overall low–normal renal function in both groups. These findings align with the results from Kok et al., which were based on eGFR assessments ≤12 months before and ≥1–2 months after CT imaging to identify long-term changes in renal function [8], although in our study, the administered iodine loads were higher for most patients and the post-procedural eGFR assessment was closer to CT imaging with a median of 20 days. Both studies did not necessarily capture all cases of contrast-induced nephropathy, which has been defined differently, but is mainly considered to occur 24 to 72 h after CM administration and to be mostly non-permanent [26]. The causative role of iodinated CM in acute kidney injury has also been questioned [26,27,28]. Nonetheless, Iacovelli et al. identified low-osmolar CM as an independent risk factor for acute kidney injury as well as 1-year mortality after TAVI and concluded that iso-osmolar CM should be considered as an alternative, especially for high-risk patients [29].

A limitation of our study is the relatively small sample size combined with a non-randomized design. However, participation in the prospective study using the CM-reduced protocol was offered to all patients referred for pre-TAVI CT and thus mitigated allocation bias. The findings are further limited by not allowing for a quality comparison of conventional images at 120 kV_p_ to images acquired using lower peak tube potentials. This drawback is due to DLCT requiring high tube potentials to capture the spectral image data that this study relies upon. Another minor limitation concerns the ROI placement in the iliac and femoral vessels, which comprised only the right side. Both sides are common access pathways for TAVI, and we do not expect this to relevantly influence our results. Lastly, we did not specifically assess the depiction of the aortic valve and annulus, which is another key element of pre-TAVI imaging. Nonetheless, the finding of sufficient attenuation in the aortic root implies a good depiction of the aortic valve complex as long as ECG gating is used.

A positive side effect of reduced CM usage may be the lower associated financial cost, which lends additional relevance from a healthcare and societal perspective. This was the primary objective of previous studies on CM reduction [30,31]. Furthermore, supply chain disruptions such as in the recent COVID-19 pandemic, which included shortages in CM supply, can be sustained better if the required amount is lowered [32].

## 5. Conclusions

Reduced iodine loads of 14–24.5 gI with BMI adjustment can provide an image quality sufficient for pre-TAVI vascular assessment in conjunction with a spectral detector CT scanner. Not being inferior to established scanning protocols with higher iodine loads, they are a feasible alternative for further reducing the required dosage of intravenous contrast agents in cardiovascular imaging. Additional studies with larger and randomized samples should be conducted in the future.

## Figures and Tables

**Table 1:** Patient characteristics. Data are presented as counts (with percentages), mean ± standard deviation, or median (minimum–maximum), depending on distribution. SCM: standard contrast media volume. RCM: reduced contrast media volume.

Parameter	SCM	RCM
Sample size (*n*)	50	50
Gender (female)	12 (24%)	22 (44%)
Age (years)	80 ± 6	83 ± 7
Height (cm)	171 ± 8	168 ± 7
Weight (kg)	77 ± 15	73 ± 16
BMI (kg/m^2^)	25 (18–40)	25 (18–45)
<23 (*n*)	11	17
23–30 (*n*)	30	25
>30 (*n*)	9	8

**Table 2:** Parameters for acquisition and reconstruction of the cardiac scan with retrospective ECG-gating and the subsequent aortoiliofemoral scan. The parameters were identical for patients from both groups.

Parameter	Cardiac	Aortoiliofemoral
Acquisition		
Mode	Low-pitch spiral	High-pitch spiral
ECG gating	Retrospective	–
Direction	Out (caudocranial)	In (craniocaudal)
Beam collimation (mm)	64 × 0.625	
Peak tube voltage (kVp)	120	
Dose modulation	On (“Cardiac”)	On (“3D Modulation”)
Tube current (mAsref)	600	130
Rotation time (s)	0.27	0.4
Pitch	0.2	1.234
Delay (s)	6 following bolus tracking (≥110 HU)	26 following bolus tracking (≥110 HU)
Reconstruction		
Cardiac phase	Best systolic, diastolic at 75% of R-R interval	–
Orientation	Axial	
Slice thickness (mm)	0.67	3.0
Increment (mm)	0.335	1.5
Algorithm	IMR Level 1	
Kernel	Cardiac Routine	Routine

**Table 3:** Attenuation values, contrast-to-noise ratios, and signal-to-noise ratios from CI, as well as attenuation values from VMI at selected energy levels. Data are presented per vessel segment as median (minimum–maximum) per the predominantly non-normal data distributions as well as corresponding *p* values (bold denotes statistical significance). CI: conventional images. VMI: virtual monoenergetic images. SCM: standard contrast media volume. RCM: reduced contrast media volume.

Series	Parameter Vessel Segment	SCM	RCM	*p* Value
	**Attenuation (HU)**			
	Ascending aorta	333 (226–651)	277 (130–335)	**<0.001**
	Aortic arch	342 (203–696)	279 (136–355)	**<0.001**
	Descending aorta	316 (221–614)	269 (177–331)	**<0.001**
	Abdominal aorta	208 (110–350)	152 (75–270)	**<0.001**
	Common iliac artery	242 (124–1170)	183 (77–290)	**<0.001**
	Common femoral artery	290 (109–679)	203 (84–331)	**<0.001**
	**Contrast-to-noise ratio**			
	Ascending aorta	11 (4–30)	10 (4–19)	0.66
	Aortic arch	14 (6–42)	12 (4–29)	0.08
**CI**	Descending aorta	10 (4–28)	9 (3–18)	0.63
	Abdominal aorta	8 (2–20)	5 (2–12)	**0.001**
	Common iliac artery	12 (3–56)	9 (2–24)	**0.01**
	Common femoral artery	20 (4–38)	12 (4–31)	**0.003**
	**Signal-to-noise ratio**			
	Ascending aorta	13 (5–33)	13 (5–23)	0.88
	Aortic arch	16 (7–46)	15 (6–34)	0.20
	Descending aorta	12 (5–32)	12 (3–22)	0.97
	Abdominal aorta	10 (5–24)	9 (4–16)	**0.002**
	Common iliac artery	15 (5–59)	12 (5–29)	**0.03**
	Common femoral artery	19 (4–50)	14 (5–33)	**0.03**
	**Attenuation (HU)**			
	Ascending aorta	735 (443–1556)	568 (222–721)	**<0.001**
**VMI** **50 keV**	Aortic arch	755 (382–1617)	561 (236–799)	**<0.001**
	Descending aorta	712 (422–1314)	549 (329–717)	**<0.001**
	Abdominal aorta	422 (191–795)	284 (116–563)	**<0.001**
	Common iliac artery	469 (199–1207)	330 (115–560)	**<0.001**
	Common femoral artery	530 (137–1423)	363 (128–612)	**<0.001**
	**Attenuation (HU)**			
	Ascending aorta	605 (371–1269)	469 (187–593)	**<0.001**
**VMI** **55 keV**	Aortic arch	619 (319–1320)	463 (199–654)	**<0.001**
	Descending aorta	583 (354–1076)	456 (275–592)	**<0.001**
	Abdominal aorta	356 (164–652)	239 (101–466)	**<0.001**
	Common iliac artery	389 (172–989)	278 (101–465)	**<0.001**
	Common femoral artery	443 (124–1170)	314 (112–508)	**<0.001**

**Table 4:** Differences in attenuation values between virtual monoenergetic imaging at selected energy levels and conventional imaging. Data are presented as median differences in attenuation values (HU) of VMI at 50 keV and 55 keV to CI as well as corresponding *p* values (bold denotes statistical significance). CI: conventional images. VMI: virtual monoenergetic images. SCM: standard contrast media volume. RCM: reduced contrast media volume.

Group	Vessel Segment	Median Diff. VMI 50 keV to CI (HU)	*p* Value	Median Diff. VMI 55 keV to CI (HU)	*p* Value
	Ascending aorta	+407	**<0.001**	+276	**<0.001**
	Aortic arch	+402	**<0.001**	+268	**<0.001**
**SCM**	Descending aorta	+396	**<0.001**	+269	**<0.001**
	Abdominal aorta	+215	**<0.001**	+147	**<0.001**
	Common iliac artery	+227	**<0.001**	+150	**<0.001**
	Common femoral artery	+250	**<0.001**	+161	**<0.001**
	Ascending aorta	+287	**<0.001**	+184	**<0.001**
	Aortic arch	+274	**<0.001**	+176	**<0.001**
**RCM**	Descending aorta	+284	**<0.001**	+190	**<0.001**
	Abdominal aorta	+132	**<0.001**	+87	**<0.001**
	Common iliac artery	+148	**<0.001**	+96	**<0.001**
	Common femoral artery	+157	**<0.001**	+103	**<0.001**

## Data Availability

The datasets presented in this article are not readily available because of privacy considerations regarding the individuals whose examinations were analyzed. Requests to access anonymized and otherwise limited datasets should be directed to the corresponding author.

## Abbreviations

CI	conventional images
CNR	contrast-to-noise ratio
CT	computed tomography
DLCT	dual-layer detector CT
eGFR	estimated glomerular filtration rate
FOV	field of view
GFR	glomerular filtration rate
SD	standard deviation
SNR	signal-to-noise ratio
TAVI	transcatheter aortic valve implantation
TAVR	transcatheter aortic valve replacement
VMI	virtual monoenergetic (monochromatic) images
